# Deep learning-based respiratory muscle segmentation as a potential imaging biomarker for respiratory function assessment

**DOI:** 10.1371/journal.pone.0306789

**Published:** 2024-07-26

**Authors:** Insung Choi, Juwhan Choi, Hwan Seok Yong, Zepa Yang

**Affiliations:** 1 Department of Integrative Medicine, Major in Digital Healthcare, Yonsei University College of Medicine, Seoul, Republic of Korea; 2 Department of Radiology, Korea University Guro Hospital, Seoul, Republic of Korea; 3 Division of Pulmonary, Allergy, and Critical Care Medicine, Department of Internal Medicine, Korea University Guro Hospital, Seoul, Republic of Korea; Chung-Ang University Gwangmyeong Hospital, REPUBLIC OF KOREA

## Abstract

Respiratory diseases significantly affect respiratory function, making them a considerable contributor to global mortality. The respiratory muscles play an important role in disease prognosis; as such, quantitative analysis of the respiratory muscles is crucial to assess the status of the respiratory system and the quality of life in patients. In this study, we aimed to develop an automated approach for the segmentation and classification of three types of respiratory muscles from computed tomography (CT) images using artificial intelligence. With a dataset of approximately 600,000 thoracic CT images from 3,200 individuals, we trained the model using the Attention U-Net architecture, optimized for detailed and focused segmentation. Subsequently, we calculated the volumes and densities from the muscle masks segmented by our model and performed correlation analysis with pulmonary function test (PFT) parameters. The segmentation models for muscle tissue and respiratory muscles obtained dice scores of 0.9823 and 0.9688, respectively. The classification model, achieving a generalized dice score of 0.9900, also demonstrated high accuracy in classifying thoracic region muscle types, as evidenced by its F1 scores: 0.9793 for the pectoralis muscle, 0.9975 for the erector spinae muscle, and 0.9839 for the intercostal muscle. In the correlation analysis, the volume of the respiratory muscles showed a strong correlation with PFT parameters, suggesting that respiratory muscle volume may serve as a potential novel biomarker for respiratory function. Although muscle density showed a weaker correlation with the PFT parameters, it has a potential significance in medical research.

## Introduction

The lungs are essential organs responsible for respiration to sustain life. Diseases that impair lung function pose a significant threat to patient health. Specifically, conditions such as Chronic Obstructive Pulmonary Disease (COPD) and asthma have serious implications for a patient’s quality of life (QoL). According to World Health Organization (WHO) statistics from 2020, diseases such as COPD, lower respiratory infection, and lung cancer rank among the top 10 leading causes of death worldwide. COPD and lower respiratory infections can be particularly severe, consistently ranking as the 3rd and 4th leading causes of death, respectively [1].

A variety of respiratory diseases, such as COPD, asthma, pulmonary fibrosis, tuberculosis, bronchiectasis, and lung cancer are driven by diverse mechanisms, including lung inflammation, excessive bronchial reactions, fibrosis, infection, and structural changes. These conditions can lead to a gradual decline in respiratory function [2–5], which may reduce oxygen saturation and lead to hypoxia. This condition, in turn, can limit the energy supply to muscles, potentially causing sarcopenia [6], and trigger a cascade of health issues including impaired respiratory and cardiac functions, diminished QoL, and increased mortality rates [2, 3, 7, 8].

Patients with respiratory diseases often undergo pulmonary function tests (PFTs) to monitor their respiratory system status. PFTs include various diagnostic techniques such as spirometry, diffusing capacity tests, lung volume assessments, and bronchodilator tests [9, 10]. Although PFTs offer valuable insights into the abnormal patterns displayed in diverse respiratory diseases, their interpretation can vary owing to the choice of reference standards and the use of somewhat arbitrary cut-off values [11]. Moreover, there are instances in which patients maintain their QoL even when negative structural changes occur in the lungs, possibly because of enhanced respiratory muscle function [8, 12–15], leading to symptom relief [16]. Therefore, to achieve accurate monitoring of the respiratory system, the analysis of PFT results should incorporate not only the clinical context and reference standards [11], but also a comprehensive approach that includes the introduction of additional biomarkers to assess respiratory muscle function. Such biomarkers may encompass the volume and density of respiratory muscles and the thickness and density of the diaphragm, as measured using thoracic CT scans. These parameters have the potential to enhance the diagnosis and monitoring of respiratory diseases, because they provide valuable insights into the correlation between a decline in respiratory muscle function and progression of respiratory conditions [17]. Consequently, these parameters can play a crucial role in adjusting patient treatment plans and predicting disease prognosis, thereby providing significant information for clinical decision-making [18, 19].

A significant correlation has previously been found between skeletal muscle mass loss and pulmonary health [18, 19], particularly in patients with COPD and asthma. The American Thoracic Society/European Respiratory Society (ATS/ERS) stated in 1999 that a decline in skeletal muscle volume is the primary cause of severe disability in patients with COPD [20]. McDonald et al. observed that, as COPD progresses through the Global Initiative for Chronic Obstructive Pulmonary Disease (GOLD) stages, the area of the pectoralis muscle, part of the respiratory muscles, tends to decrease. This suggests it could be a useful noninvasive marker to assess respiratory muscle impairment [21]. Moreover, skeletal muscle loss in patients with COPD has been confirmed as an independent predictor of mortality [22]. Similarly, research on asthma patients has revealed that those with lower muscle mass tend to have more blocked airways and a higher risk of severe exacerbation [23]. Patients with higher-density respiratory muscles show reduced respiratory symptoms, such as breathlessness upon exercise [24, 25]. Therefore, the precise measurement and analysis of these respiratory muscles are vital for predicting disease prognosis and formulating appropriate treatment plans.

Computed tomography (CT) is a versatile tool used to diagnose various human diseases. One of its advantages is its capability for the quantitative volumetric assessment of the anatomy of patients, making it particularly useful for quantifying respiratory muscles. However, traditional approaches generally involve in-slice measurement techniques This process involves finding a specific cross-sectional slice using biomarkers or physiological indicators and measuring the area and distribution of Hounsfield units (HU) in that slice. Cruz-Jentoft et al. advocated this approach, suggesting that the muscle area in a single slice at the level of the third lumbar vertebra (L3) was highly correlated with the whole-body muscle mass and could thus serve as a surrogate for muscle quantification [26]. Zhi et al. indicated that the muscle area measured at the T12 could serve as a substitute for L3 and act as an independent predictor of hospital mortality among older patients with COPD admitted to intensive care units [27]. Kim et al. [7] measured the cross-sectional area of the pectoralis muscle in a single slice immediately above the aortic arch and found a moderate correlation between the pectoralis muscle area and total skeletal muscle mass.

Although single axial slice-based methods for measuring muscle mass are convenient and time-efficient, they cannot provide comprehensive three-dimensional volumetric information of the region of interest. Consequently, the quantitative metrics derived from these single-slice images may not accurately assess the organs under study.

With rapid advancements in medicine, artificial intelligence-based segmentation technologies are gaining attention, particularly in the automatic segmentation of complex muscle areas that serve as quantitative measurement standards [28–30]. Technologies that utilize CT images have been explored for various organs. For example, Hiasa et al. introduced a Bayesian U-Net model for gluteal and thigh muscle segmentation, incorporating uncertainty metrics from the training process to identify less reliable segmentation predictions [28]. Through active learning, this model refined the accuracy in these areas. Qadri et al. employed a stacked sparse autoencoder (SSAE) for spine segmentation using subdivided CT image patches to illustrate the efficacy of the SSAE in localized tasks [29]. Chen et al. presented a lung-dense attention network with residual spatial attention and gated channel attention mechanisms [30]. By assigning weights to the pulmonary regions, this model outperformed traditional segmentation methods in the lung areas.

This study aimed to develop an automated approach for the segmentation of three types of respiratory muscles (the pectoralis, erector spinae, and intercostal muscles) from CT images using artificial intelligence techniques. Furthermore, this study proposes an intuitive approach for calculating the volume and density of segmented respiratory muscles. Using the measured volume and density, the study also investigated the correlation with the PFT elements to explore the potential of respiratory function biomarkers.

## Materials and methods

### Study design

This retrospective study was conducted in compliance with the Helsinki Declaration and was approved by the Institutional Review Board (IRB) of Korea University Guro Hospital (IRB No.: 2022GR0185, 2018GR0179, and 2021GR0335). The datasets, collected from the IRB-approved research, were accessed from September 22, 2021, to June 21, 2022. Authors had no access to information that could identify individual participants during or after data collection. The need to obtain informed consent was waived by the Institutional Review Board due to the retrospective nature of the study.

The datasets used in this study included all Korean fat and muscle mass data constructed by the National Information Society Agency (NIA) [31] and anonymized thoracic CT images of patients with respiratory diseases and healthy individuals collected at Korea University Guro Hospital. A concise overview of the datasets is presented in Table 1. A detailed description of the data can be found in the section entitled "Detailed Description of the Dataset”.

**Table 1:** Summary of datasets and corresponding IRB numbers.

*Dataset ID*	*Data Provider*	*Dataset Description*	*IRB Number*
D1	NIA	Thoracic CT data with muscle tissue masks	2022GR0185
D2	KUGH	Thoracic CT data with respiratory muscle masks	2022GR0185
D3	KUGH	Thoracic CT data without masks	2018GR0179
			2021GR0335

KUGH = Korea University Guro Hospital; NIA = National Information Society Agency

**Fig 1:**
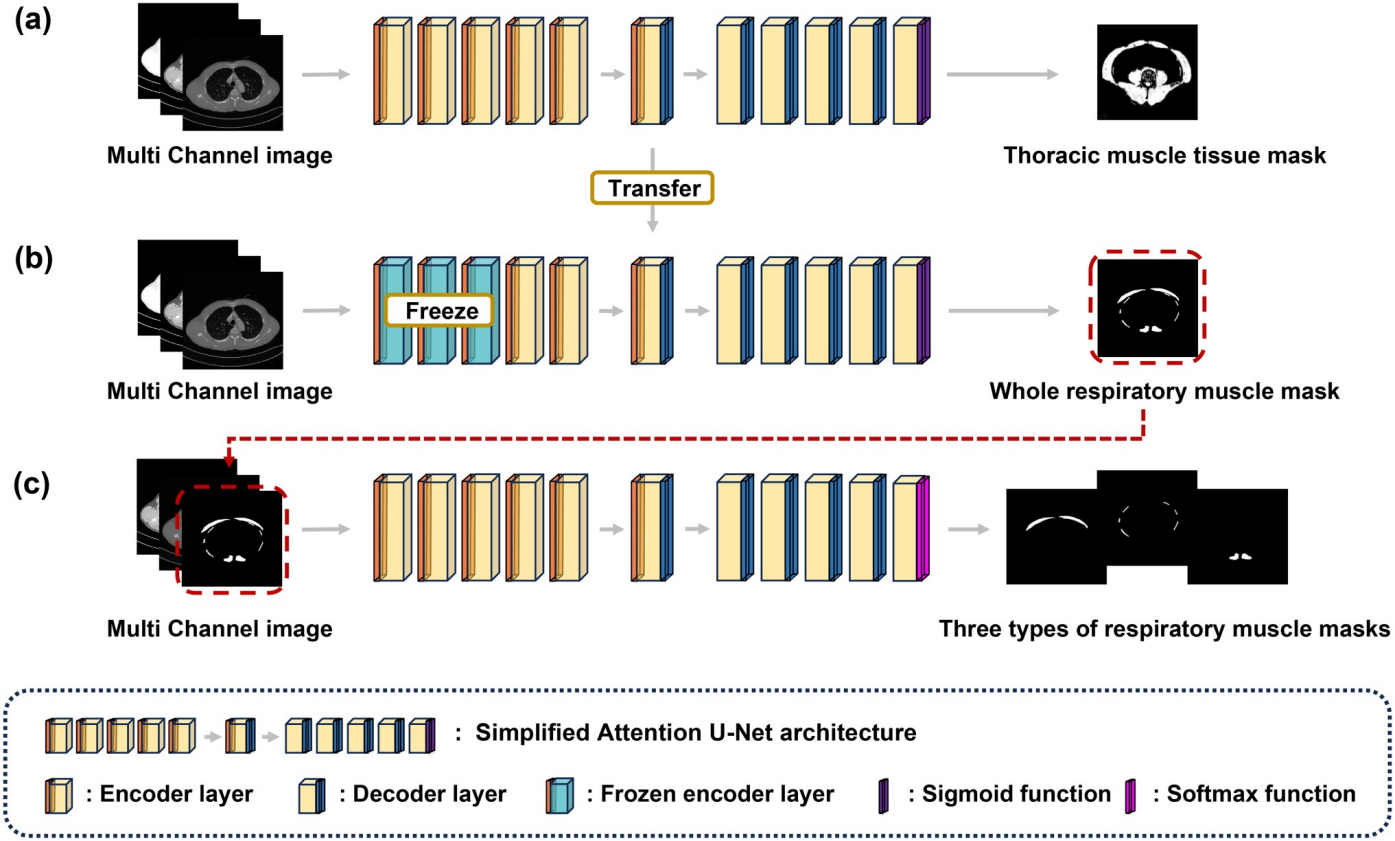
Overview of the experimental workflow. (a) The Muscle Tissue Segmentation Model. The model for segmenting all muscle tissues from thoracic CT scans. (b) Comprehensive Respiratory Muscle Segmentation Model. This model processes CT images to produce comprehensive respiratory muscle masks by fine-tuning the model depicted in (a), which includes freezing the first three layers of the encoder. (c) Respiratory Muscle Classification Model. This model classifies individual respiratory muscle masks from the Comprehensive Respiratory Muscle Mask.

The segmentation and classification of the respiratory muscles were conducted using the empirically modified Attention U-Net model, divided into the following three specialized stages: muscle tissue segmentation, respiratory muscle segmentation, and respiratory muscle classification. Fig 1 presents the workflow of this study. The performance of the model was validated using machine-learning metrics and visual assessments by radiologists. The volume and density of the respiratory muscles were calculated using pixel-based methods by analyzing the areas within the muscle regions identified on the binary masks. To examine the correlation between these measurements and PFT parameters, we employed the scipy.stats.spearmanr function from the SciPy library (version 1.9.0), utilizing the Spearman’s rank correlation method. Two NVIDIA V100 GPUs provided by NIA and an NVIDIA RTX A6000 from the Korea University Guro Hospital were utilized to train the machine-learning models. The deep learning framework employed was PyTorch version 1.10.2.

### Detailed description of the dataset

The imaging data utilized in this study comprised 512×512 pixel DICOM images and corresponding 512×512 pixel muscle tissue masks. Muscle tissue segmentation model data were derived from Korean fat and muscle mass data, previously established by the NIA, and used with ethical approval from the IRB of Korea University Guro Hospital (Table 1, Dataset ID: D1). The dataset was meticulously curated by first selecting individuals based on their daily living capabilities and primary symptoms, followed by a secondary selection of healthy individuals based on the PET/CT imaging results. A total of 3,000 individuals, whose CT data were devoid of noise and artifacts, and without any significant underlying diseases, were selected for this study. The selected data were anonymized to remove sensitive information and randomly sampled for cross-validation by two radiologists to ensure that only data with 100% agreement of specialist opinions were included. The finalized dataset was processed using an annotation tool to mask the muscle regions, which were then extracted in NII and TIF formats.

To train the model responsible for segmenting and classifying respiratory muscle areas from thoracic CT scans, we utilized CT images from Korea University Guro Hospital. This dataset, approved for use by Guro Hospital’s IRB (Table 1, Dataset ID: D2), comprised data from 202 individuals, including patients with various respiratory diseases, including COPD and asthma, and healthy individuals. Patients with severe underlying conditions or complicated muscle mass measurements were excluded from the study. The selected images were anonymized, and sensitive data were excluded and checked for compliance with the international standards of medical imaging. For the annotation process of our study, the pectoralis, erector spinae, and intercostal muscles within all CT images were manually annotated by three experienced radiologists. The quality and accuracy of the annotated masks were confirmed through a consensus reached during meetings of the three radiologists, where every mask was thoroughly reviewed and refined. Table 2 shows the detailed characteristics of the datasets.

**Table 2:** General characteristics of patient data for distribution of the respiratory muscles.

*Characteristics*	*Values*
Number of participants	202
Age (years)	68.10±10.87
Sex (F/M)	150/52
Tube voltage peak (kV)	120
Standard dose scans	55
• Reference mAs	100
Low dose scans	147
• Reference mAs	40
Automatic Exposure Control	Applied
Reconstruction kernel	Br59
Slice thickness (mm)	3
COPD diagnosed	84
Sarcopenia progressed	35
Normal patients	118

COPD = Chronic Obstructive Pulmonary Disease; mAs = milliampere-seconds

To further assess the utility and validate the performance of our respiratory muscle segmentation and classification model, we employed an additional dataset from Korea University Guro Hospital (Table 1, Dataset ID: D3). This dataset comprised CT scans from 1,674 individuals aged between 40–80 years, without pre-existing respiratory muscle masks. This facilitated the analysis of the relationship between elements of the PFT and respiratory muscles, as well as the exploration of the distribution of total respiratory muscle volume across various age and sex groups. The specific characteristics of this dataset are detailed in Table 3.

**Table 3:** General characteristics of patient data for the distribution of respiratory muscles.

*Characteristics*	*Values*	
	*Male*	*Female*
Number of participants	1170	504
Age (years)	68.25±9.65	66.76±10.43
Tube voltage peak (kV)	120	
Standard dose scans	689	256
• Reference mAs	100	
Low dose scans	481	248
• Reference mAs	40	
Automatic Exposure Control	Applied	
Reconstruction kernel	Br59	
Slice thickness (mm)	3	

mAs = milliampere-seconds

### Deep learning for respiratory muscle segmentation and classification in CT

**Fig 2:**
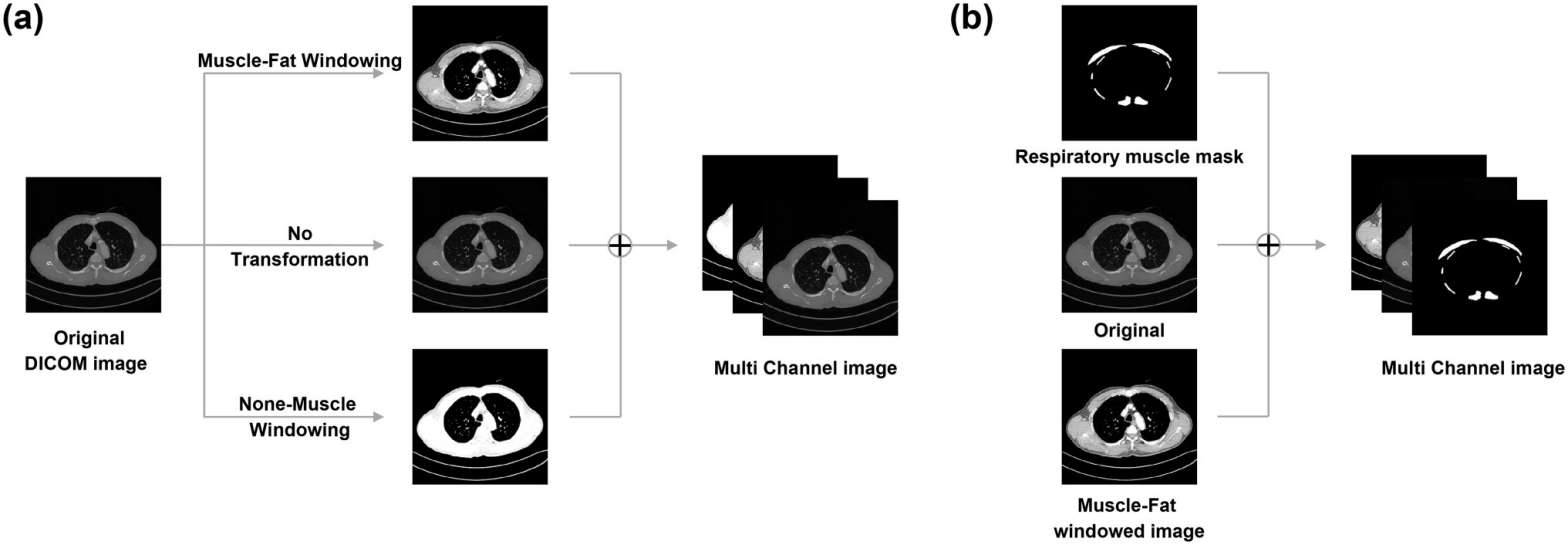
Schematic of the 3-channel image generation process. (a) Process of creating input image data for the muscle tissue segmentation and respiratory muscle segmentation models (b) Process of Creating Input Image Data for Respiratory Muscle classification model.

The muscle tissue segmentation model in this study was trained using a NIA dataset to segment all the muscle tissues within the thoracic region. Image preprocessing was conducted to enhance the muscle distribution clarity and increase the robustness of the model. This included creating a 3-channel input image by combining images based on distinct HU ranges, as used by radiologists in clinical settings for muscle localization [32–34]. These channels comprised the original DICOM images (-1024 HU to 3071 HU), images considering the HU ranges for muscles and fat (-250 HU to 150 HU), and those below the muscles and fat HU range (below 250 HU). The methodology is illustrated in Fig 2a. Additionally, image augmentation techniques, such as horizontal flipping and rotation, were applied without compromising the intrinsic features of the CT images.

**Fig 3:**
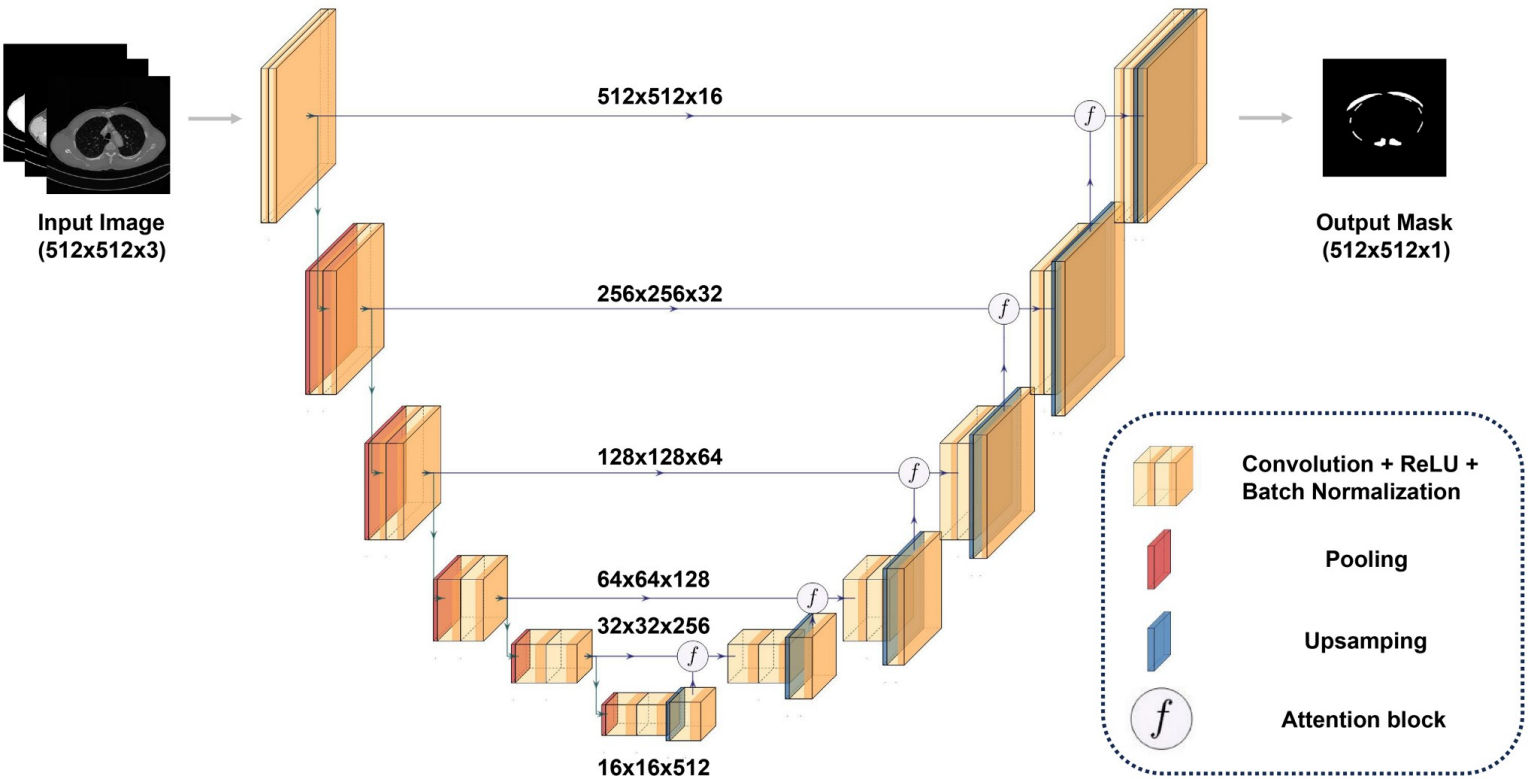
Attention U-Net architecture. The unit model was designed with six layers of each encoder/decoder, and the attention gates were joined within the decoder. These gates operate in conjunction with the feature maps from both the encoder and decoder, which are transmitted via skip connections.

The muscle tissue segmentation model employed the Attention U-Net architecture (Fig 3), which is known for its effectiveness in medical image segmentation [35]. This architecture integrates attention gates to focus on target areas and enhance the segmentation precision. A sigmoid activation function was used in the output layer for binary classification of the pixels (Fig 1). The training data were divided in an 8:2 ratio for training and testing, using dice loss as the loss function. For model training, the Adam optimizer was used with epochs set to 100 and a batch size of 8. The initial learning rate was set to 0.0001, and if there was no improvement in dice loss for three consecutive epochs, the learning rate was reduced by 70%. Post-processing steps, including noise removal from the respiratory muscle masks using OpenCV, were performed to eliminate connected components below a certain size.

Chest CT images from Korea University Guro Hospital were used to train the model to segment and classify respiratory muscle areas from thoracic CT scans. Of the 202 patients, 150 had complete labeling of the three types of respiratory muscles (pectoralis, intercostal, and erector spinae muscles), each accompanied by three mask images per DICOM image. To address the lack of labels in the remaining data, an active learning approach was adopted during the training process of the respiratory muscle segmentation model [36]. Initially, the muscle tissue segmentation model was fine-tuned using data from 150 labeled patients. To preserve pre-learned weights and biases for common features, we froze the first three layers of the encoder. This allowed the model to learn high-level semantic information like the structure of the respiratory muscles. Subsequently, the fine-tuned model predicted the respiratory muscle areas in subsets of the 52 unlabeled patients. Experts then refined the predicted masks, using the corresponding probability maps as a reference. Once the new masks were obtained, the accumulated data were used to retrain the model from scratch, thereby enhancing the performance of the respiratory muscle segmentation model. This process was repeated until all unlabeled data were processed. Data preprocessing techniques, dataset division, loss functions, optimizer, learning rate and post-processing of prediction data consistently followed the methods used in muscle tissue segmentation model training, because of the common goal of binary muscle segmentation. Epochs and batch size were set at 50 and 4, respectively. The respiratory muscle classification model categorized the segmented results of the comprehensive respiratory muscle model into three classes. Although the training data remained the same as in the respiratory muscle segmentation model, the input image processing differed. The input image comprised a uniformly segmented respiratory muscle mask, an image considering the HU range for muscles and fat (-250 HU to 150 HU), and an original DICOM image (-1024 HU to 3071 HU), forming a multichannel structure (Fig 2b). The input labels were assigned unique pixel values for different muscle classes to differentiate them from the segmentation model. Pixel values in overlapping muscle areas prioritized the pectoralis and erector spinae muscles over the intercostal muscles. The training data were split in an 8:2 ratio for training and testing. The same Attention UNet architecture was employed for muscle classification, which was expedited by the proven efficacy of the architecture on the same dataset. However, a softmax activation function was used at the output layer for multiclass classification, and generalized dice loss was employed during training [37, 38]. The optimizer, learning rate, epoch, and batch size were applied identically to those used in the respiratory muscle segmentation.

### Model performance evaluation

The segmentation and classification performance of the proposed model were evaluated in both numerical and empirical perspective ways. First, machine learning metrics such as the Dice Similarity Coefficient score and the Generalized Dice Similarity Coefficient score were applied to evaluate the segmentation model, while precision, recall, and F1 score metrics were employed to assess the classification model. Second, experienced radiologists visually assessed the segmentation results by assigning quantitative scores ranging from 1 to 10, based on criteria such as slice- and volume-based accuracy and under/over-segmentation.

### Additional experimental analysis

After consulting respiratory specialists, closely related PFT indicators were selected for correlation analysis between respiratory muscle volume and density measurements and PFT variables. For the correlation analysis, we utilized the PFT information acquired within three months of the CT scan date and conducted a Spearman’s rank correlation. The volume and density of the respiratory muscles were calculated using the predictive masks generated by the segmentation and classification models presented in this paper. The volume was determined based on mask pixels and slice thickness, while the density was calculated using the HU values corresponding to each pixel.

Furthermore, the distribution of the total volume of respiratory muscles across age groups and gender was investigated to assess the diverse research applicability of the proposed muscle measurement methodology.

## Results

### Respiratory muscle segmentation and classification

**Fig 4:**
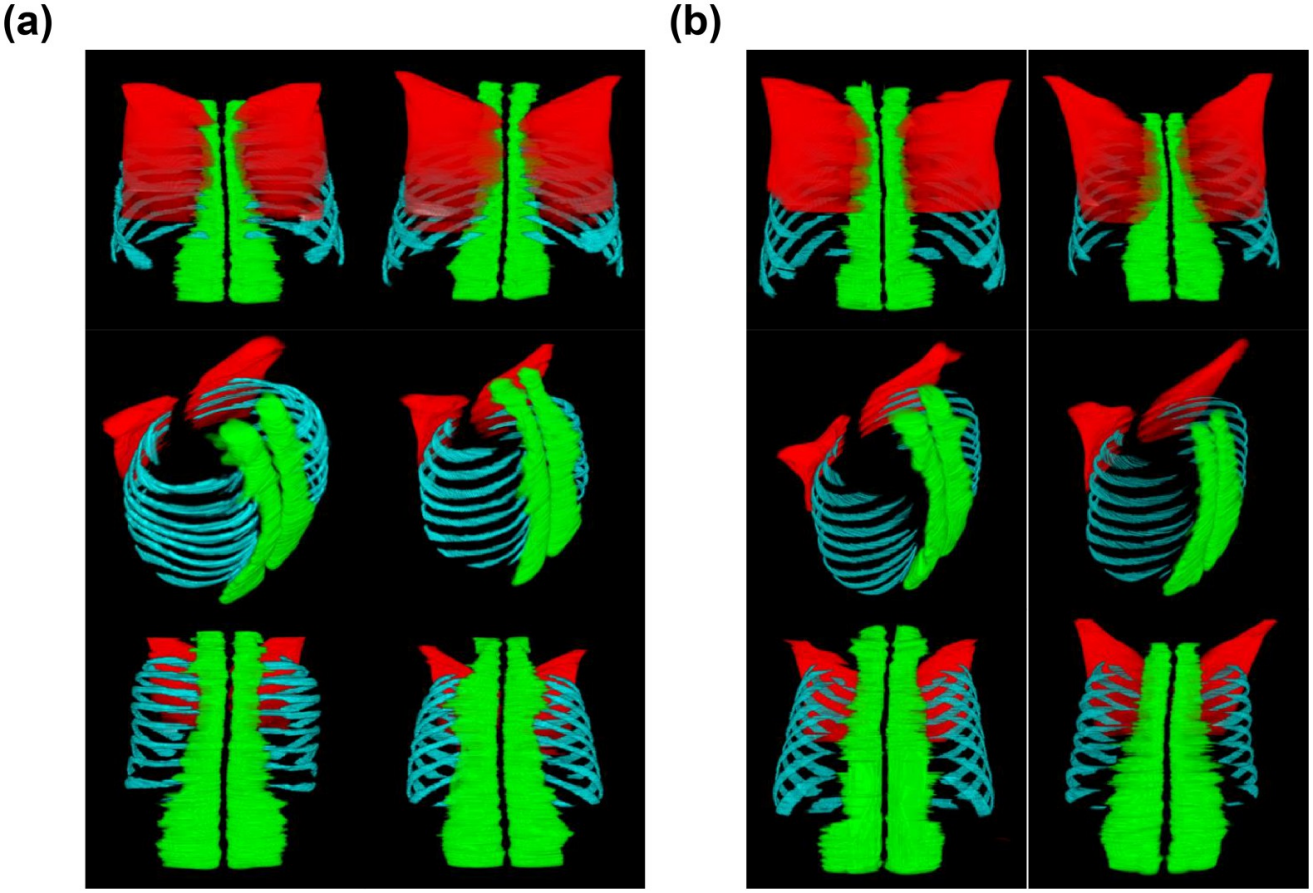
Model predictions of three-dimensional respiratory muscle segmentation. (Pectoralis Major Muscles: Red, Intercostal Muscles: Blue, Erector Spinae Muscles: Green): (a) Example of Successful Segmentation, (b) Example of Inadequate Segmentation. Compared to a case of successful segmentation, there is a relative lack of continuity between the Intercostal Muscles and the Erector Spinae Muscles.

**Fig 5:**
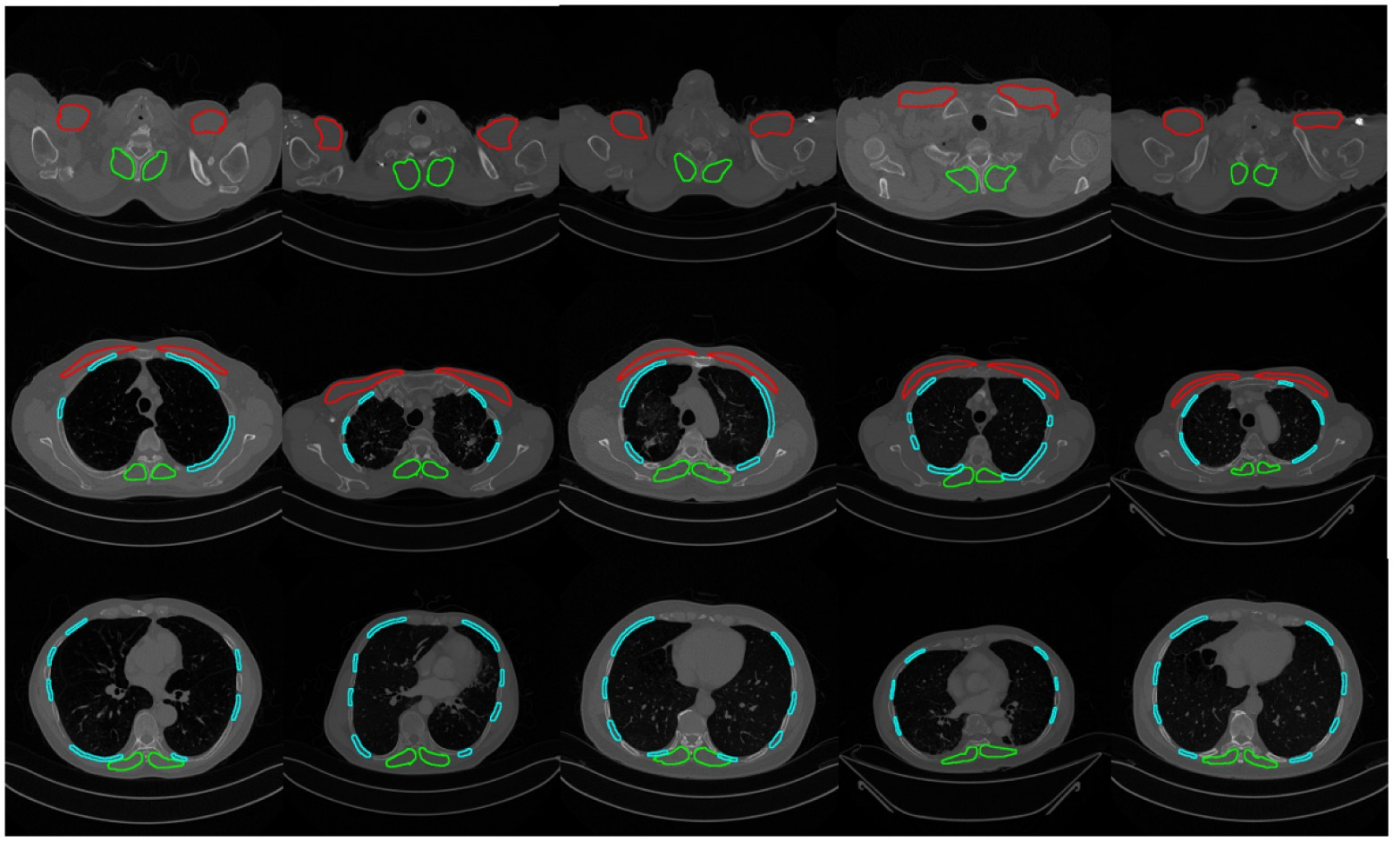
Muscle segmentation results on axial slices of thoracic CT scans. In each image, the upper section represents the ventral aspect, while the lower section depicts the dorsal aspect. The first row illustrates the upper part of the thorax, with the second and third rows progressively moving towards the lower part. (Pectoralis Muscles: Red, Intercostal Muscles: Blue, Erector Spinae Muscles: Green).

The segmentation results for the respiratory muscles obtained using the proposed methodology are shown in Figs 4 and 5. The dice scores for the muscle tissue segmentation model and respiratory muscle segmentation model on the test set were 0.9823 and 0.9688, respectively, with dice loss values of 0.0177 and 0.0312, respectively. The generalized dice score for the three types of respiratory muscle classification models was 0.9930, with a generalized dice loss of 0.0070. For the classification model, the dice scores for the pectoralis, erector spinae, and intercostal muscles were 0.9794, 0.9976, and 0.9839, with corresponding loss values of 0.0206, 0.0024, and 0.0161, achieving a generalized dice score of 0.9900. The model’s classification performance, indicated by F1 scores, was 0.9793 for the pectoralis muscle, 0.9975 for the erector spinae muscle, and 0.9839 for the intercostal muscle. Details related to the model’s classification performance metrics are presented in Table 4.

**Table 4:** Classification performance metrics for each respiratory muscle: Precision, recall, F1 score, and overall accuracy.

*Muscles*	*Precision*	*Recall*	*F1 Score*	*Accuracy*
Pectoralis muscle	0.9881	0.9748	0.9793	0.9822
Erector spinae muscle	0.9995	0.9957	0.9975	
Intercostal muscle	0.9761	0.9929	0.9839	

According to visual evaluations by radiology experts, the accuracy score of the model for respiratory muscle segmentation ranged from 8 to 9 on a 10-point scale, indicating generally favorable assessments. Regarding the segmentation errors, the rate of insufficient muscle segmentation was higher than the converse rate. Considering the expert interpretations and the overall score for all criteria, the segmentation performance for the intercostal muscles was comparatively lower; however, the proposed respiratory muscle segmentation method ultimately demonstrated good performance. The results of the visual evaluation are shown in Table 5.

**Table 5:** Results of visual assessment.

Segmentation assessment criteria	Muscle		
	Pectoralis	Erector spinae	Intercostal
Segmentation accuracy			
Slice segmentation accuracy	9.5	9.8	9.2
Volume segmentation accuracy	8.3	8.1	7.2
Anatomical detail accuracy	8.8	9.2	7.5
Segmentation Errors			
**Under-segmentation**	**1.5**	**2.2**	**2.7**
Over-segmentation	1.3	1.0	0.8
Consistency with expert opinion	9.0	9.2	8.5
**Overall quality**	**8.5**	**8.3**	**7.4**

### Additional experimental analysis

Further analyses investigated the correlations between the estimated volumes and densities of the pectoralis, erector spinae, and intercostal muscle regions with parameters from the PFT. These parameters, outlined in Table 6, include forced vital capacity (FVC), inspiratory capacity (IC), vital capacity (VC), forced expiratory volume in one second (FEV1), total lung capacity (TLC), forced expiratory volume in six seconds (FEV6), and the diffusing capacity of the lungs for carbon monoxide (DLCO). The selection of PFT indicators for inclusion was based on advice from pulmonary specialists, ensuring their significant relevance to respiratory conditions. The volumes and densities of these three types of respiratory muscles were calculated based on the dataset provided by Korea University Guro Hospital (referenced as Table 1, Dataset ID: D3). Results of these calculations are presented in Table 7.

**Table 6:** Results of Spearman rank correlation analysis.

Variable	Volume			Density		
	PM	ESM	ICM	PM	ESM	ICM
n	1,674	1,674	1,674	1,674	1,674	1,674
Mean	289.28	478.58	148.90	47.82	47.99	28.85
SD	139.98	149.53	42.21	5.84	6.20	12.11
FVC	.81	.75	.72	.62	.55	.46
IC	.79	.73	.67	.63	.61	.47
VC	.79	.73	.66	.63	.63	.48
FEV1	.77	.71	.61	.63	.48	.48
TLC	.75	.68	.67	.55	.48	.40
FEV6	.74	.67	.65	.57	.56	.40
DLCO	.71	.68	.59	.59	.64	.48

ESM = Erector spinae Muscle; ICM = Intercostal Muscle; PM = Pectoralis Muscle; SD = standard deviation

**Table 7:** Quantitative analysis of respiratory muscle volume and density based on the proposed models.

*Characteristics*	*Values*	
	*Male*	*Female*
Number of participants	1170	504
Respiratory Muscle Quantitative Metrics		
Pectoralis muscle Volume (cc)	343.01±123.87	158.40±57.70
Erector spinae muscle Volume (cc)	500.47±141.46	387.42±97.18
Intercostal muscle Volume (cc)	154.26±40.27	125.07±28.75
Whole muscle Volume (cc)	997.75±261.45	670.89±140.48
Pectoralis muscle Density (HU)	48.65±5.26	45.40±6.56
Erector spinae muscle Density (HU)	48.42±5.63	46.45±6.87
Intercostal muscle Density (HU)	28.43±12.05	30.93±12.69
Whole muscle Density (HU)	45.99±5.59	43.72±7.00

HU = Hounsfield unit

Factors within the respiratory muscles that exhibited a strong correlation with the PFT components were the volumes of the pectoralis and erector spinae muscles, especially the volume of the pectoralis muscles, which showed strong correlations with all selected PFT components. Overall, the density of the pectoralis muscles and volume of the intercostal muscles exhibited moderate correlations with the PFT components, whereas the density of the erector spinae muscles and intercostal muscles showed relatively weaker correlations. From the standpoint of respiratory muscle elements, PFT parameters that displayed strong correlations mainly included FVC, IC, and VC, whereas the correlations with FEV1, TLC, FEV6, and DLCO varied depending on the specific respiratory muscle factors.

**Fig 6:**
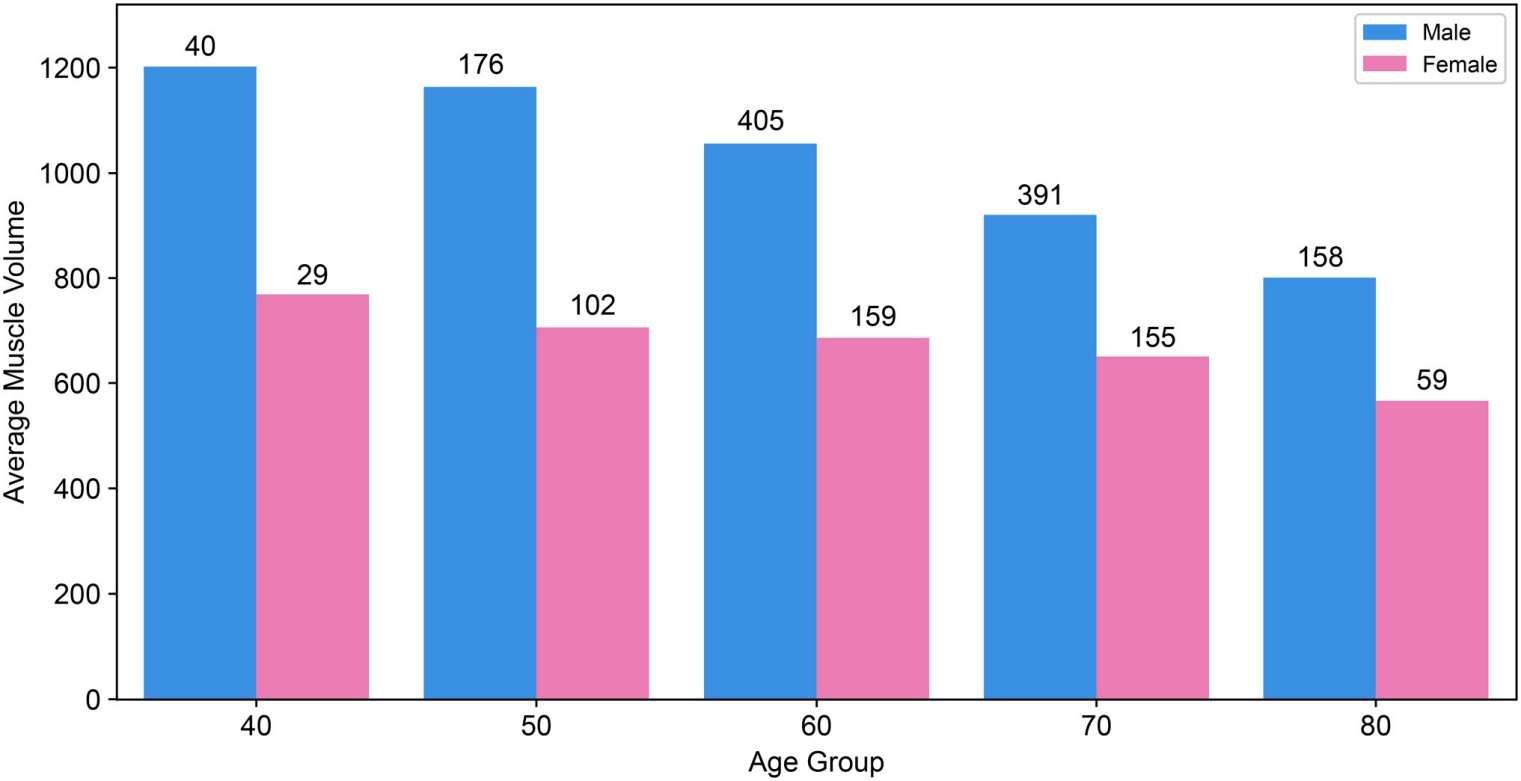
Distribution of average respiratory muscle volume by age group and sex. (Male: Blue, Female: Pink).

**Fig 7:**
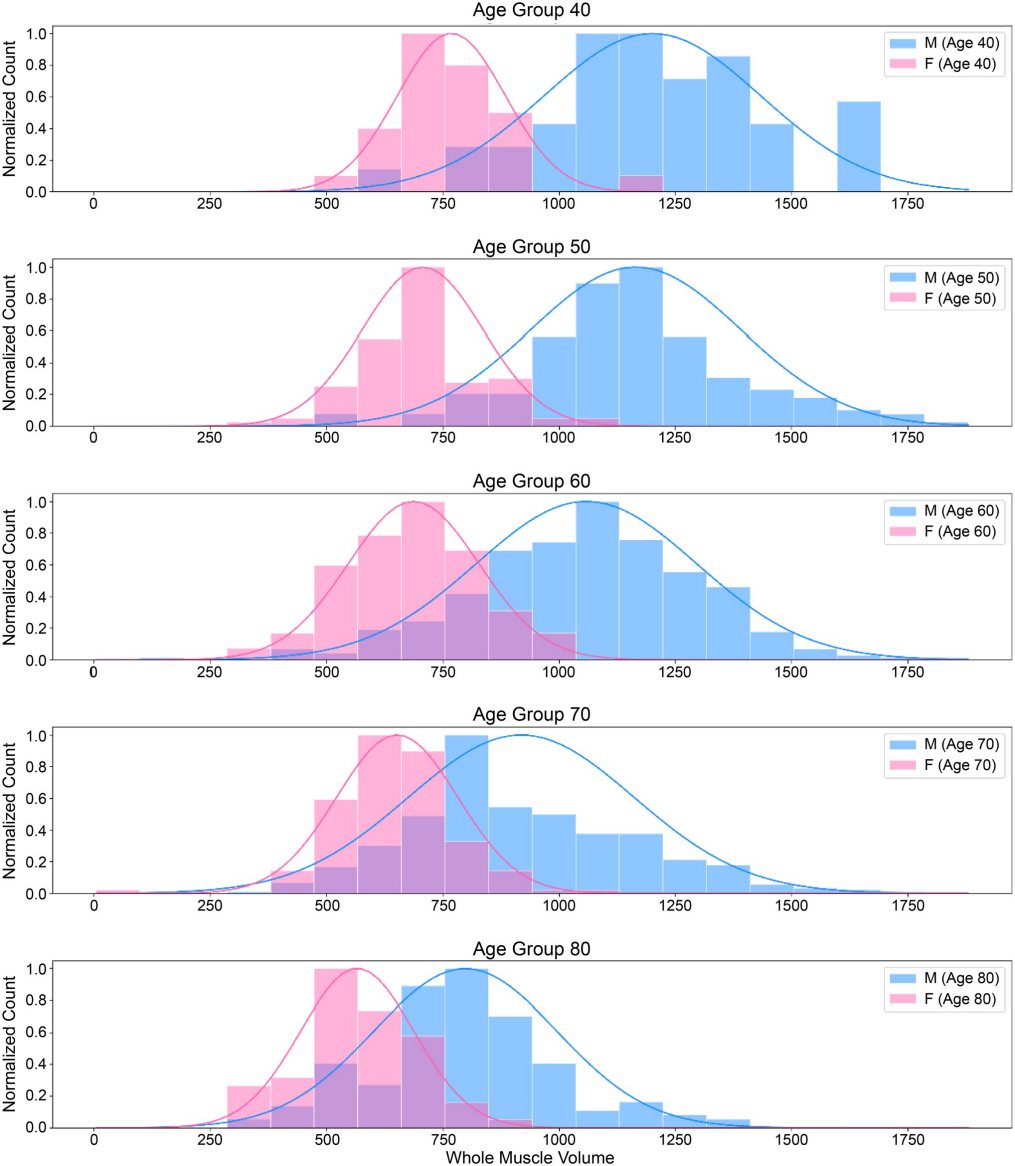
Distribution of respiratory muscle volume by age group and sex. (Male: Blue, Female: Pink).

Utilizing the total volume data of respiratory muscles detailed in Table 7, Figs 6 and 7 depict the distribution across various age groups (40–80 years) and sexes, respectively. As the age increased, the volume of the respiratory muscles decreased. Within each age group, males exhibited a greater total volume of respiratory muscles than females.

## Discussion

In this study, we developed a deep-learning-based respiratory muscle segmentation and classification model capable of quantifying the volume and density of respiratory muscles from CT images. For the accurate analysis of the characteristics and structure of respiratory muscles, in CT images, it is necessary to acquire information from all slices. This is essential because features, including muscle morphology or the muscle-to-fat ratio, are likely to vary across the entire set of slices. Relying solely on representative slices for muscle measurement does not consider such heterogeneity, potentially leading to an inaccurate reflection of the overall characteristics of the tissue. Therefore, the volume and density measurement techniques of the respiratory muscles proposed through our model serve as a meaningful approach for more accurate quantification of muscle attributes.

We selected the Attention U-Net for respiratory muscle segmentation because of its superior performance over other state-of-the-art methods in prior research that focused on pectoralis muscle segmentation in CT-based studies [39]. To address the scarcity of labeled data in the medical domain, we chose to employ a muscle tissue segmentation model that was trained on a large dataset. This strategic choice allowed us to significantly enhance the performance of our respiratory muscle segmentation by fine-tuning the muscle tissue segmentation model with a smaller scale dataset related to respiratory muscle segmentation.

In this study, two reasons prompted the adoption of separate segmentation and classification models to individually segment the three types of respiratory muscles in the CT images. First, we aimed to improve performance by constructing specialized models for each task. The respiratory muscle segmentation model was optimized to distinguish between the muscle and background, thereby reducing the complexity of the problem. The respiratory muscle classification model focuses solely on a finer categorization within the respiratory muscles. In doing so, we managed to mitigate the complexity of the problem and enhance the model performance. Second, the introduction of a modular model design ensures the functional independence of each model, thus providing a flexible foundation for its application in various medical research contexts. For instance, in situations requiring the segmentation or classification of other thoracic muscles, the modular design of separate models can enhance research efficiency. Indeed, our goal extends beyond respiratory muscles, with the aim of encompassing the segmentation and classification of a broad range of thoracic muscles, which motivated our choice for a modular model design approach.

The experimental analysis focused on exploring the correlations between PFT parameters and the respiratory muscles’ volume and density, as well as muscle volume variations across gender and age groups, aiming to identify patterns and trends rather than statistical significance. In a study examining the correlation between PFT parameters and the volume and density of respiratory muscles, respiratory muscle volume showed strong correlations with PFT parameters including FVC, IC, VC, FEV1, TLC, FEV6, and DLCO. PFT parameters play a pivotal role in the diagnosis of a wide range of respiratory diseases, particularly in evaluating respiratory function. Therefore, our analysis indicates the potential of using the quantitative volume of the respiratory muscles as a biomarker for the assessment of respiratory function. This finding offers a novel approach to overcome the limitations of existing PFT methodologies.

The correlation between respiratory muscle density and PFT results was relatively weak. However, Nachit et al. reported a significant association between intramuscular fat deposition and mortality risk [40]. Therefore, we anticipate that respiratory muscle density could be utilized in future studies to assess the prognosis in patients with various diseases, including respiratory illnesses.

If the proposed algorithm is used to obtain sex-specific, year-wise, and age-wise muscle distributions over a designated period, it could facilitate the exploration of diseases that correlate with muscle distribution trends. As such, we believe that the accumulated muscle data could contribute to the discovery of new biomarkers and aid in other clinical studies related to trends in muscle distribution.

Despite the advantages of the methods and findings proposed in this study, some limitations should be highlighted. One significant challenge was the 3 mm slice thickness of our CT dataset, hindering the accurate segmentation of fine structures such as the diaphragm. The loss of continuity and clarity in sagittal plane imaging necessitated the exclusion of the diaphragm from our study scope despite its critical role in respiration. In future work, we aim to develop rule-based algorithms for diaphragm segmentation using thinner CT slices to overcome this limitation. Additionally, this study’s reliance on a dataset from a specific hospital may limit the generalizability of our findings. Variations in imaging protocols across CT scanners could also impact model performance. The observed weak correlation between muscle density and PFT parameters also underscores the need for further validation. Finally, focusing only on static images omits the dynamic nature of muscle function during respiration, suggesting that future studies should incorporate multimodal data for a more comprehensive analysis.

## Conclusion

In this study, we developed a deep-learning-based model for the segmentation and classification of respiratory muscles, and further verified its capability to measure the volume and density of respiratory muscles precisely in CT images. Although the model demonstrated limitations in being optimized for a specific protocol, the quantified volume of the respiratory muscles showed a correlation with various PFT elements related to respiratory capacity. Additionally, the density of the respiratory muscles suggests high applicability in the field of medical research. These quantified metrics for respiratory muscles have the potential to serve as new biomarkers that can be applied in the diagnosis and treatment of respiratory diseases, as well as foundational data for subsequent research across various diseases and conditions.
